# Assessment of prognostic risk of death in patients with multiple myeloma based on CD184 and CD269: A retrospective analysis

**DOI:** 10.5937/jomb0-56987

**Published:** 2025-09-05

**Authors:** Hong Chen, Yuan Zhao, Zhiyu Zhang, Yan Xie, Mulan Jin

**Affiliations:** 1 Beijing Chaoyang Hospital, Capital Medical University, Department of Pathology, Beijing, 100043, China

**Keywords:** multiple myeloma, CD184, CD269, prognosis, multipli mijelom, CD184, CD269, prognoza

## Abstract

**Background:**

This study aimed to analyze the expressions of CD184 and CD269 in patients with newly diagnosed multiple myeloma (MM) in China and explored their potential association with prognosis.

**Methods:**

This retrospective study recruited 100 patients with MM and 113 healthy controls who attended Beijing Chaoyang Hospital Shijingshan District between June 2020 and December 2023. The association between CD184 and CD269 expression and patient survival outcomes was assessed using univariable and multivariable Cox proportional hazards regression analyses, alongside Kaplan-Meier survival curves.

**Results:**

Both CD184 and CD269 mRNA were elevated in MM patients compared to controls (P<0.05). By qualitative analysis, it was seen that both CD184+ and CD269 patients had a reduced prognostic overall survival (OS) (P<0.05). Similarly, detection of CD184 and CD269 expression was effective in predicting prognostic mortality in patients.

**Conclusions:**

CD184 and CD269 may serve as valuable prognostic markers in MM patients.

## Abstract (sr)

**Uvod:**

Ova studija je imala za cilj analizu ekspresije CD184 i CD269 kod pacijenata sa novodijagnostikovanim multiplim mijelomom (MM) u Kini i istraživanje njihove potencijalne povezanosti sa prognozom.

**Metode:**

Ova retrospektivna studija je obuhvatila 100 pacijenata sa MM i 113 zdravih kontrolnih pacijenata koji su bili na lečenju u bolnici Peking Chaoyang, okrug Shijingshan, u periodu od juna 2020. do decembra 2023. Povezanost ekspresije CD184 i CD269 sa ishodima preživljavanja pacijenata procenjena je korišćenjem univarijabilne i multivariable regresione analize proporcionalnih rizika po Koksovom modelu, kao i Kaplan-Majerovim krivama preživljavanja.

**Rezultati:**

Ekspresija mRNK CD184 i CD269 bila je povećana kod pacijenata sa MM u poređenju sa kontrolnim pacijentima (P<0,05). Kvalitativna analiza je pokazala da su pacijenti sa CD184+ i CD269 imali smanjeno ukupno preživljavanje (OS) (P<0,05). Takođe, detekcija ekspresije CD184 i CD269 se pokazala efikasnom u predviđanju prognoze smrtnog ishoda kod pacijenata.

**Zaključak:**

CD184 i CD269 mogu da budu značajni prognozički markeri kod pacijenata sa MM.

## Introduction

Multiple myeloma (MM) is a plasma cell neoplastic disease characterized by the malignant proliferation of monoclonal plasma cells in the bone marrow, the presence of monoclonal immunoglobulin in the blood or urine, and related end-organ damage (including hypercalcemia, renal failure, anemia, and bone lesions) [1] [2] [3] and is the second most common hematological malignancy in older adults, accounting for 1% of all cancers [2] [3]. MM is characterized by a high heterogeneity, and the survival of the patients varies from months to years [2] [3]. Although the available markers and staging systems can reflect the condition and prognosis of most patients, more simple prognostic biomarkers are still needed. In addition, with the advent of the recent immunotherapy paradigm in cancer treatment, detecting new immune indicators at initial diagnosis could be of clinical significance for the treatment and prognosis of MM patients.

CD184, also named C-X-C chemokine receptor 4 (CXCR4), is a member of the G protein-coupled seven transmembrane receptor protein superfamily. It is abnormally expressed in different solid tumors, and its high expression is associated with tumor metastasis and poor patient prognosis [4]. CD184 is considered a potential therapeutic target for malignant tumors and is important for improving the progression of malignant tumors [5]. It is known that a high number of CD184+ cells in autologous hematopoietic stem cell transplantation (auto-HSCT) is associated with a higher mortality risk [6]. However, the relation of CD184 with prognosis in MM remains poorly understood. CD269, also known as the B cell maturation antigen (BCMA) and as tumor necrosis factor receptor superfamily member 17, is expressed only on plasma cells and plasmacytoid dendritic cells in normal human tissues [7]. CD269 maintains the longterm survival of plasma cells in the bone marrow [8]. Currently, studies have demonstrated that elevated CD269 expression is associated with a poorer prognosis in MM [9]. Recently, in a study on active myeloma by Jakubikova J et al. [10], they mentioned that B-cell signaling regulators represented by CD184 and CD269 largely determine the progression of the patient’s disease. These findings suggest that CD184 and CD269 may be equally important for MM, but few studies have reported the value of combined testing of CD184 and CD269 for disease assessment in MM.

Therefore, this study will provide a preliminary analysis of the clinical significance of CD184 and CD269 in MM and further analyze the relationship between CD184, CD269 and the prognosis of MM patients. These results will provide a new reference for future diagnosis and treatment of MM, thus improving the prognosis of MM patients [11] [12].

## Materials and methods

### Study design and patients

This observational study enrolled patients at the Department of Hematology, Shijingshan District of Beijing Chaoyang Hospital between June 2020 and December 2023. This study was approved by the Medical Research Ethics Committee of Beijing Chaoyang Hospital, Capital Medical University. Due to the retrospective nature of the study, the ethics committee waived the requirement for individual consent.

We calculated the sample size needed for the study by using the PASS software (NCSS, USA), 100 patients with MM and 113 patient cases with nonhematologic diseases with normal bone marrow function confirmed by bone marrow aspiration examination in the same period (control group) were included in this study (Table 1). The MM patients all received chemotherapy with bortezomib, lenalidomide, and cyclophosphamide at our hospital.

**Table 1:** Baseline information of MM patients and controls.

	MM patients (n=100)	Control group (n=113)	t/χ^2^	P
Sex (male/female)	56/44	68/45	2.557	0.110
Age, years, median (range)	69.7 (43–88)	72.6 (40–79)	1.873	0.063
Typing, n (%)				
IgG type	50 (50.0%)	-		
Type IgA	22 (22.0%)	-		
Light chain type	26 (26.0%)	-		
Other	2 (2.0%)	-		
Hemoglobin (g/L)	92.6 (45–183)	118.7 (99–143)	4.472	0.001
Lactate dehydrogenase (U/L)	218.7 (96.3–768.4)	166.7 (117.1–365.2)	2.834	0.006
β2-microglobulin (mg/L)	5.34 (2.12–30.58)	1.99 (1.42–2.70)	5.225	0.001
DS stage, n (%)				
I+II	21 (21.0%)	-		
III	79 (79.0%)	-		
R-ISS stage, n (%)				
I+II	28 (28.0%)	-		
III	72 (72.0%)	-		
Cytogenetics, n (%)				
High risk	31 (31.0%)	-		
Standard risk	69 (69.0%)	-		
Cytological type, n (%)				
Mature type	72 (72.0%)	-		
Naive/primitive type	13 (13.0%)	-		
Intermediate type	15 (15.0%)	-		

The inclusion criteria were 1) met the diagnostic criteria for MM in the Chinese Guidelines for the Diagnosis and Treatment of Multiple Myeloma (2022 revision) (11), 2) > 18 years old, and 3) complete dataset. The exclusion criteria were 1) combined with other types of blood diseases, 2) with infectious or immune diseases, or 3) combined with other types of malignant tumors. All study participants underwent bone marrow biopsy and bone marrow flow cytometry for staging and disease characterization as per routine procedures during the study period.

### Data collection and outcomes

Bone marrow cells were collected from all the study subjects after admission to the hospital, and the expression of CD184 and CD269 was detected by PCR. Total cell RNA was extracted by TRlzol (Thermo Fisher, USA) and reverse transcribed to cDNA according to the kit (Thermo Fisher, USA) instructions for the amplification reaction of CD184 and CD269. The reaction conditions were as follows: 94°C for 4 min, 94°C for 15 s, 60°C for 40 s, 72°C for 30 s, for a total of 40 cycles. The primer sequences were designed and constructed by Jiangsu Saisofei Biotechnology Co., Ltd (Table 2), and the 2^-ΔΔCt^ method was used to calculate the relative expression of CD269 and CD184.

**Table 2:** Sequence of primers.

	Forward Primer	Reverse Primer
CD184	5’-AGA GCA GAC AGC GAG GAC AT-3’	5’-GGA GTG TGA CAG TTT GGA GTG-3’
CD269	5’-CTG GCT GCT GCT GCT GCT-3’	5’-GGA GGA GGA GGA GGA GGA-3’
GAPDH	5’-GGA GCG AGA TCC CTC CAA AAT-3’	5’-GGC TGT TGT CAT ACT TCT CAT GG-3’

Bone marrow flow cytometry data were collected for CD184, CD269. Antigen positivity was definedas 20% of the cells positive for the antigen [12]. Accordingly, the patients were designed into CD184+ vs CD184- and CD269+ vs CD269-.

FISH cytogenetic data were collected from the charts regarding IgH translocation, 1q21 amplification, 17p- (P53 deletion), and 1p32 deletion. Those with q21 amplification, 17 p- (P53 deletion), P53 mutation, or t (4; 14), t (14; 16), t (14; 20) were defined as high genetic risk, while those without the above genetic changes were defined as standard risk [13].

The patients were followed up by reviewing the electronic medical records. The follow-up was censored on January 1, 2024. The outcomes of this study were overall survival (OS). OS was defined as the time from diagnosis to death from any cause or censored on the last follow-up.

### Statistical analysis

SPSS 27.0 (IBM, USA) was used for statistical analysis. The continuous data were tested using the Shapiro-Wilk test. Those with a normal distribution were presented as means ± standard deviations and analyzed using Student’s t-test; otherwise, they were presented as median (range) and analyzed using the Mann-Whitney U-test. Categorical data were expres sed as n (%) and analyzed using the chi-squared test or Fisher’s exact test. Diagnostic values were analyzed using the receiver operating characteristic (ROC) curve. The Kaplan-Meier method was used to draw the survival curves of OS, and the curves were compared using the log-rank test. Univariable regression analyses were performed, and the variables with *P*<0.05 were included in the multivariable Cox proportional hazards regression analysis to explore the factors independently associated with OS in patients with MM. Two-sided *P*-values <0.05 were defined as statistically significant.

## Results

### Diagnostic efficacy of CD184 and CD269 in MM

**Figure 1:**
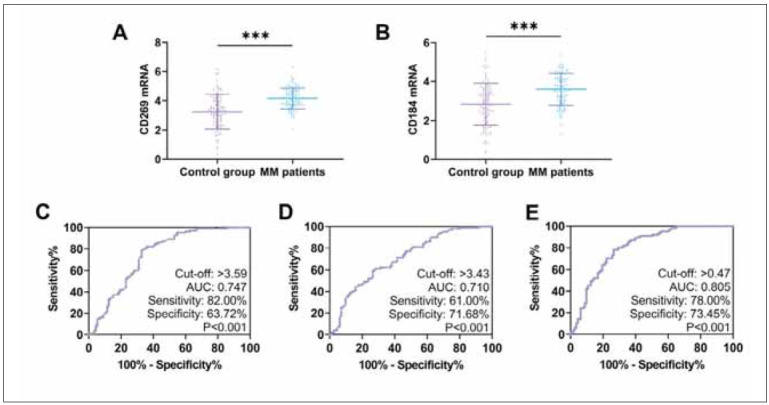
Diagnostic efficacy of CD184 and CD269 in MM.<br>(A) Comparison of CD269 mRNA in MM patients and controls. (B) Comparison of CD184 mRNA in MM patients and controls. (C)ROC curve for CD269 diagnosis of MM occurrence. (D) ROC curve for CD184 diagnosis of MM occurrence. (E) ROC curve of the combined CD269 and CD184 assay for the diagnosis of MM occurrence

First, we compared the results of quantitative analysis of CD184 and CD269 between the two studygroups. The results showed that both CD184 and CD269 mRNA expression were higher in MM patients than in the control group (*P*<0.05). The results of correlation factor analysis showed that both CD184 and CD269 affected the independent risk factors of MM occurrence (*P*<0.001). By ROC curve, we found that both CD184 and CD269 mRNA showed excellent diagnostic effect on the occurrence of MM (CD184: AUC=0.710, CD269: AUC=0.747). And when the two were combined, the sensitivity for diagnosing MM occurrence was 78.00% and the specificity was 73.45% (*P*<0.001, AUC=0.805) (Figure 1 and Table 3).

**Table 3:** Relationship between CD269 and CD184 and MM. B: regression coefficient; S.E.: standard error; OR: odds ratio; 95%CI: 95% confidence interval.

	B	S.E.	Wald	P	OR	95%CI
CD269	0.951	0.189	25.165	<0.001	2.587	1.785–3.751
CD189	0.777	0.175	19.817	<0.001	2.175	1.545–3.062

### Relationship between CD184, CD269 and pathologic features of MM

73 patients (73.0%) were CD184+, and 27 patients (27.0%) were CD184-. There were no significant differences in sex, age, M protein type, hemoglobin (Hb), lactate dehydrogenase (LDH), Durie- Salmon (DS) stage [14], Revised International Staging System (R-ISS) stage [15], and myeloma cell morphology between the two groups (all *P*>0.05), but the β2-microglobulin (MG) levels (*P*=0.044) and the proportion of high-risk cytogenetics (*P*=0.033) were significantly higher in the CD184+ group than that in CD184- group.

Among the MM 100 patients, 68 (68.0%) were CD269+, and 32 (32.0%) were CD269-. There were no significant differences in sex, age, M protein classification, LDH, β2-MG, DS stage, R-ISS stage, high-risk cytogenetics, and myeloma cell morphology between the two groups (all *P*>0.05). The Hb levels in the CD269+ group were significantly lower than in the CD269- group (*P*=0.004) (Table 4).

**Table 4:** Characteristics of the patients according to the expression of CD184 and CD269 in bone marrow tissue of patients with MM. DS: Durie-Salmon; R-ISS: revised International Staging System.

Variables	CD184+<br>(n=73)	CD184-<br>(n=27)	t/χ^2^	P	CD269+<br>(n=68)	CD269-<br>(n=32)	t/χ^2^	P
Sex (male/female)	42/31	14/13	0.258	0.611	41/27	15/17	1.590	0.207
Age, years, median<br>(range)	68.2 (43–85)	71.3 (49–88)	0.214	0.428	72.7 (55–82)	66.8 (43–88)	0.382	0.573
Typing, n (%)								
IgG type	37 (50.7%)	13 (48.1%)	0.953	0.813	35 (51.5%)	15 (46.9%)	0.472	0.925
Type IgA	16 (21.9%)	6 (22.2%)			15 (22.1%)	7 (21.9%)		
Light chain type	18 (24.7%)	8 (29.6%)			17 (25.0%)	9 (28.1%)		
Other	2 (2.7%)	0 (0.0%)			1 (1.5%)	1 (3.1%)		
Hemoglobin (g/L)	90.4<br>(51–169)	94.2 (45–183)	0.382	0.705	86.3 (45–178)	101.6<br>(59–183)	-2.910	**0.004**
Lactate dehydrogenase<br>(U/L)	234.9<br>(112.2–768.4)	201.3<br>(96.3–729.7)	0.841	0.406	238.3<br>(122.2–668.7)	199.5<br>(96.3–768.4)	1.203	0.232
β2-microglobulin<br>(mg/L)	6.56<br>(2.47–30.58)	3.78<br>(2.12–22.56)	2.081	**0.044**	6.85<br>(2.12–30.58)	5.46<br>(2.78–22.40)	0.236	0.893
DS stage, n (%)								
I+II	16 (21.9%)	5 (18.5%)	0.137	0.711	15 (22.1%)	6 (18.8%)	0.144	0.705
III	57 (78.1%)	22 (81.5%)			53 (77.9%)	26 (81.3%)		
R-ISS stage, n (%)								
I+II	20 (27.4%)	8 (29.6%)	0.049	0.825	17 (25.0%)	11 (34.4%)	0.949	0.330
III	53 (72.6%)	19(70.4%)			51 (75.0%)	21 (65.6%)		
Cytogenetics, n (%)								
High risk	27 (37.0%)	4 (14.8%)	4.530	**0.033**	24 (35.3%)	7 (21.9%)	1.832	0.176
Standard risk	46 (63.0%)	23 (85.2%)			44 (64.7%)	25 (78.1%)		
Cytological type, n (%)								
Mature type	54 (74.0%)	18 (66.7%)	0.545	0.761	47 (69.1%)	25 (78.1%)	2.897	0.235
Naive/primitive type	9 (12.3%)	4 (14.8%)			8 (11.8%)	5 (15.6%)		
Intermediate type	10 (13.7%)	5 (18.5%)			13 (19.1%)	2 (6.3%)		

### COX analysis affecting prognostic survival in MM patients

The variables assessed included age, Hb, LDH, β2-MG, DS stage, R-ISS stage, High-Risk Cytogenetics, Immature myeloma cell type (Naive/primitive type and Intermediate type), myeloma cell CD184+ and myeloma cell CD269+. In the univariate model, immature myeloma cell type andmyeloma cell CD269+ and CD184+ were found to signi cantly affect OS. Further multivariate analysis revealed that myeloma cell CD269+ and CD184+ were signi cantly related to OS (Table 5).

**Table 5:** Univariable and multivariable analysis affecting OS in MM patients. Hb: haemoglobin; LDH: lactate dehydrogenase; β2-MG: β2-microglobulin; DS: Durie-Salmon; R-ISS: revised International Staging System; Immature plasma cell type (Naive/primitive type and Intermediate type).

Factors	OS			
	Univariate analysis		Multivariate analysis	
	*HR (95%CI)*	*P*	*HR (95%CI)*	*P*
Age	2.671 (0.929–5.542)	0.509		
Hb	0.990 (0.219–4.466)	0.989		
LDH	1.363 (0.271–5.852)	0.707		
β2-MG	0.693 (0.175–2.749)	0.602		
DS Stage III	1.820 (0.159–6.875)	0.631		
R-ISS Stage III	2.848 (0.571–6.204)	0.202		
High-Risk	1.436 (0.225–4.144)	0.702		
Immature type	2.482 (1.571–3.718)	**0.032**	1.512 (1.109–2.928)	0.406
CD184+	2.500 (1.478–6.642)	**0.007**	1.874 (1.164–4.997)	**0.026**
CD269+	4.783 (2.278–10.822)	**0.009**	2.341 (1.221–7.837)	**0.022**

### Relationship between CD184, CD269 and prognostic survival in MM patients

**Figure 2:**
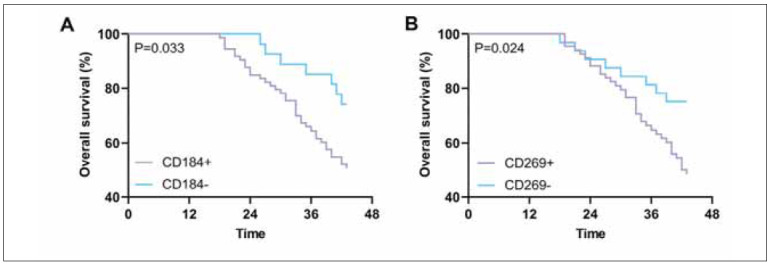
Survival analysis according to CD184 and CD269 expression in patients with MM.<br>(A) Kaplan-Meier survival curves of overall survival (OS) in patients with multiple myeloma according to the CD184+ and CD184- groups. (B). Kaplan-Meier survival curves of OS in patients with multiple myeloma according to the CD269+ and CD269- groups.

Compared with CD184- patients, CD184+ patients had shorter prognostic OS (*P*<0.05). Similarly, CD269- patients had a higher prognostic OS than CD269+ patients (*P*<0.05) (Figure 2).

### Predictive value of CD184 and CD269 for prognostic mortality in MM

**Figure 3:**
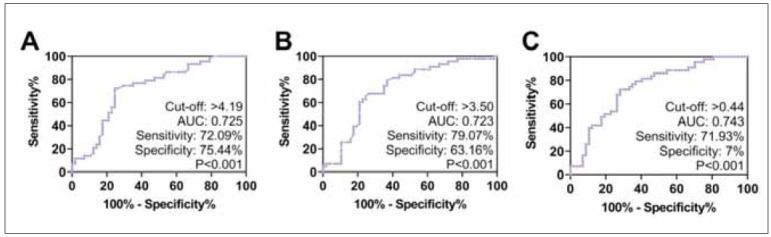
Predictive value of CD184 and CD269 for prognostic mortality in MM<br>(A) ROC curves for prognostic death in MM diagnosed by CD269. (B) ROC curves for prognostic death in MM diagnosed by CD184. (C) ROC curve of CD269 in combination with CD184 for the diagnosis of prognostic mortality in MM.

Finally, we analyzed the predictive value of CD184 and CD269 for prognostic mortality in MMusing ROC curves. The results showed that CD184 had a predictive sensitivity of 79.07% and a specificity of 63.16% for prognostic death in MM (*P*<0.001, AUC=0.723). In contrast, the sensitivity and specificity of CD269 in predicting prognostic death in MM patients were 72.09% and 75.44%, respectively (*P*<0.001, AUC=0.725). However, in the prediction of prognostic death, the combined test of CD184 and CD269 did not show a significant advantage (*P*<0.001, AUC=0.743) (Figure 3).

## Discussion

The results indicate that CD184 and CD269 may be useful markers of prognosis in patients with MM. CD269+ and CD184+ was independently associated with poor OS. First, we found that CD184 was elevated in MM patients. CD184 is normally expressed on T and B lymphocytes, monocytes, macrophages, neutrophils, and eosinophils and is expressed by hematopoietic stem/progenitor cells residing in bone marrow niches. In MM, the expression of CD184 can induce the movement and settlement of MM cells in the bone marrow microenvironment, promote their homing and localization, and promote the growth and migration of MM [15] [16]. CD184 plays a key role in the invasion, metastasis, and drug resistance of myeloma [17] and solid tumors [18] [19] [20] [21]. CD184 can enhance the activation of osteoclasts and promote the growth of bone tumors [22]. promote osteolysis and degradation of extracellular matrix, leading to detachment of myeloma cells from the primary tumor site, entry into the bloodstream, and distant organ metastasis [23] [24].

The present study showed that the serum levels of β2-MG in the CD184+ group were significantly increased; β2-MG is an important prognostic indicator of MM [25]. When MM is active, a large amount of β2-MG can be released into the blood, and its level can reflect the patient’s renal function and tumor cell activity [26]. The risk stratification of Mayo myeloma stratification and risk-adapted therapy (mSMART) 3.0 [27] is one of the key factors in determining the prognosis of MM, and the proportion of high-risk patients in the risk stratification of the CD184+ group is higher. Therefore, CD184+ patients have a worse prognosis in terms of renal dysfunction and risk stratification. CD184+ was independently associated with a poorer OS, highlighting the importance of evaluating CD184 expression in patients with MM. On the other hand, the relation of CD184 expression with prognosis could be context-dependent since a study showed that CD184 expression in harvest cells was associated with engraftment in patients undergoing hematopoietic cell transplantation [6], while another study showed that CD184 and CD34 double staining couldpredict the cell mobilization outcome [28]. Zheng et al. [29] reported that CD184 was expressed in plasma cell myeloma and could be used as a diagnostic and therapeutic target. Those results warrant further study on the role of CD184 in MM and hematological malignancies.

CD269 is mainly expressed in B cells and plays a crucial role in the process of B cell proliferation, survival, maturation, and differentiation into plasma cells. CD269 is specifically expressed on plasmolysis cells and differentiated plasma cells but not on naive B cells, memory B cells, CD34+ positive hematopoietic stem cells, or other normal tissues [30]. CD269 is consistently expressed in MM cells, and its expression in malignant plasma cells is significantly higher than in normal plasma cells [31]. Serum CD269 levels gradually increase from healthy people to untreated monoclonal gammopathy, smoldering myeloma, and then MM, and its concentration is positively correlated with disease status and prognosis [32]. Serum CD269 is elevated in MM patients and can predict clinical outcomes, so it is regarded as a biomarker to monitor disease progression. The present study showed that the expression rate of CD269 in MM patients was 68.0%. The Hb levels of CD269+ patients were significantly lower than in CD269- patients, suggesting more obvious anemia. CD269+ patients have an increased risk of prognostic death, which confirms the relationship between CD269 and poor prognosis in MM. Nevertheless, it supports the idea that CD269 expression is associated with poor patient outcomes. The expression level of CD269 in monoclonal plasma cells of bone marrow of patients with MM varies greatly (0–100%), and the proportion is about 8.6% in CD269-negative patients, regardless of new diagnosis, relapse, progression, or treatment [33]. The relative expression of CD269 in bone marrow tissue is related to the clinical stage and survival time of MM but not to sex, age, cytological type, and recurrence, indicating that the increased expression of CD269 may promote the malignant progression of MM and lead to poor prognosis [34].

The multivariable Cox regression analysis showed CD184+ cells increased the risk of disease progression or death by 1.874 times, and CD269+ increased 2.314 times. These findings suggest that detecting CD184 and CD269 can serve as useful prognostic indicators for patients, and the results of the analysis of the ROC curve confirm this view.

This study preliminarily explored the expression and clinical significance of CD184 and CD269 in MM, which has certain clinical practice guidance but also has certain limitations. Due to the small sample size, some test results may not be consistent with those reported in the literature. Secondly, whether there could be an improper selection of antibody fluorescence channels or redefinition of positive threshold in the detection of CD269 needs to be further optimized in future work. Thirdly, the retrospective nature of the study limited the data that could be analyzed to the data available in the patient charts. In this regard, we should subsequently increase the number of cases studied and extend the follow-up period so as to improve the representativeness and comprehensiveness of the results. At the same time, we need to add more assays (e.g., immunohistochemistry, Western blot, etc.) to validate the results of this study. Finally, we also need to carry out in vitro tests as soon as possible to confirm the exact mechanism of action of CD184 and CD269 on MM.

## Conclusion

The detection of CD184 and CD269 by flow cytometry may be used as indicators to evaluate the prognosis of MM patients. These markers provide simple prognostic biomarkers and reliable laboratory evidence for subsequent treatment of patients.

## Dodatak

### Acknowledgements

None.

### Funding

No funding was provided for this article.

### Availability of data and materials

The data that support the findings of this study are available from the corresponding author upon reasonable request.

### Conflict of interest statement

All the authors declare that they have no conflict of interest in this work.
